# Milk Exosomal miR-27b Worsen Endoplasmic Reticulum Stress Mediated Colorectal Cancer Cell Death

**DOI:** 10.3390/nu14235081

**Published:** 2022-11-29

**Authors:** Elisa Martino, Anna Balestrieri, Luigi Mele, Celestino Sardu, Raffaele Marfella, Nunzia D’Onofrio, Giuseppe Campanile, Maria Luisa Balestrieri

**Affiliations:** 1 Department of Precision Medicine, University of Campania Luigi Vanvitelli, Via L. De Crecchio 7, 80138 Naples, Italy; 2 Food Safety Department, Istituto Zooprofilattico Sperimentale del Mezzogiorno, Via Salute 2, 80055 Portici, Italy; 3 Department of Experimental Medicine, University of Campania Luigi Vanvitelli, Via Luciano Armanni 5, 80138 Naples, Italy; 4 Department of Advanced Clinical and Surgical Sciences, University of Campania Luigi Vanvitelli, Piazza Miraglia, 80138 Naples, Italy; 5 Department of Veterinary Medicine and Animal Production, University of Naples Federico II, Via F. Delpino 1, 80137 Naples, Italy

**Keywords:** milk, exogenous miRNA, endoplasmic reticulum stress, colorectal cancer

## Abstract

The relationship between dietary constituents and the onset and prevention of colorectal cancer (CRC) is constantly growing. Recently, the antineoplastic profiles of milk and whey from Mediterranean buffalo (*Bubalus bubalis*) have been brought to attention. However, to date, compared to cow milk, the potential health benefits of buffalo milk exosome-miRNA are still little explored. In the present study, we profiled the exosomal miRNA from buffalo milk and investigated the possible anticancer effects in CRC cells, HCT116, and HT-29. Results indicated that buffalo milk exosomes contained higher levels of miR-27b, miR-15b, and miR-148a compared to cow milk. Mimic miR-27b transfection in CRC cells induced higher cytotoxic effects (*p* < 0.01) compared to miR-15b and miR-148a. Moreover, miR-27b overexpression in HCT116 and HT-29 cells (miR-27b^+^) induced apoptosis, mitochondrial reactive oxygen species (ROS), and lysosome accumulation. Exposure of miR-27b^+^ cells to the bioactive 3kDa milk extract aggravated the apoptosis rate (*p* < 0.01), mitochondrial stress (*p* < 0.01), and advanced endoplasmic reticulum (ER) stress (*p* < 0.01), via PERK/IRE1/XBP1 and CHOP protein modulation (*p* < 0.01). Moreover, GSK2606414, the ER-inhibitor (ER-i), decreased the apoptosis phenomenon and XBP1 and CHOP modulation in miR-27b^+^ cells treated with milk (*p* < 0.01 vs. miR-27b^+^+Milk), suggesting the ER stress as a cell-death-aggravating mechanism. These results support the in vitro anticancer activity of 3kDa milk extract and unveil the contribution of miR-27b in the promising beneficial effect of buffalo milk in CRC prevention.

## 1. Introduction

Milk and dairy products are naturally characterized by the presence of a large number of functional molecules, including amino acids, vitamins, fats, oligosaccharides, and proteins, which play several biological roles [1,2,3,4,5]. The antioxidant, antimicrobial, antihypertensive, and immunomodulatory properties of milk-derived bioactive peptides have been extensively reported [6,7], as well as the biological functions of bovine milk lipidomics, related to a lower risk for cardiovascular disease and type 2 diabetes [8]. The peculiar milk bioactivity is also maintained in dairy products and by-products, as recently reported [9]. The health properties of milk from Mediterranean buffalo (*Bubalus bubalis*) are further ascribed to its peculiar content in L-carnitine, betaines, and short-chain acyl-carnitines [10,11], which can be improved by innovative breed and breeding techniques [12,13]. Buffalo milk-derived bioactive molecules protected the endothelial cells against in vitro hyperglycemia-induced cell damage [13,14], displayed antineoplastic activities in squamous carcinoma cells, promoted cell cycle arrest, mitochondrial ROS accumulation, and apoptosis [13,15]. In human colorectal cancer (CRC) cells, milk betaine caused cell cycle modulation, mitochondrial apoptosis, redox state alteration, and epigenetic modification [16,17]. In addition to milk, whey, a dairy by-product with a functional metabolomic profile close to milk in terms of betaines and short-chain acyl-carnitines [12], showed bioactive properties in CRC models. In LoVo, SW480, HCT116, and HT-29 cells, milk whey triggered metabolic alteration and apoptosis, with more pronounced effects on HCT116 and HT-29 cells [18]. The in vitro observations were supported by HCT116 xenograft mouse model of CRC, showing the activation of the necroptotic pathway via increased RIPK1, RIPK3, and MLKL protein expression levels [19].

Milk is a known source of exosome-packaged microRNA (dietary miRNAs), small noncoding RNAs containing about 19–22 nucleotides, which negatively regulate the expression of their targeted genes at the post-transcriptional level by driving the degradation of target mRNAs [20,21]. Milk exosomes have attracted considerable attention as a therapeutic approach in different diseased tissues [22] given their content in bioactive cargos and miRNAs, able to influence different health outcomes [23]. Exosomal miRNAs are involved in several processes affected by biogenesis and exosome contents and play an important regulatory role in a wide range of biological and pathological pathways, including cancer development [24,25,26]. MiRNAs contained in milk exosomes are related to nervous system pathways and brain development during early life [27]. MiR181a-5p showed antiatherogenic effects by inhibiting NF-κB activation and, in turn, vascular inflammation [28]. In addition, milk-derived miR-31-5p encouraged cell growth and migration of endothelial cells in vitro and stimulated angiogenesis and enhanced diabetic wound healing in a mouse model [29].

Nutrition alters endogenous miRNA expression, and exogenous functional miRNAs assumed with diet are transferred and bioavailable to organisms [26]. MiRNAs from cow milk modulate crucial pathways in osteogenesis, nervous, and brain development, as well as regulation of B and T lymphocytes, macrophage activation, and modulation of the adaptative immune response [23]. Milk-derived miRNAs are also able to prevent the onset of rheumatoid arthritis and to downregulate the expression of several colitis-associated miRNAs, thus improving the macroscopic colitis histopathological scores [30,31,32]. Buffalo milk-derived exosomes are correlated with immune response and metabolic processes [33,34]. However, the role of milk-derived miRNAs in cancer is still contrasting. Acting as oncomirs, milk-derived miR-21 and miR-155 have been related to breast cancer progression, enhancing tamoxifen resistance, metastasis formation, and worse prognosis [35], while miR-125b is involved in prostate xenograft cancer by targeting tumorigenic pathways, as p53, PI3K/AKT/mTORC1, ERBB2, and Wnt [36,37]. On the contrary, epidemiological studies highlighted that milk intake is related to a lower incidence of CRC, showing that consumption of dairy products decreases the risk of cardiovascular disease and CRC [38,39].

CRC, the predominant global health burden in developed countries, ranking third place in terms of incidence and the second in mortality worldwide, is classified among the cancers most closely associated with diet, also based on age and hereditary factors [40,41]. To date, the knowledge of food influence on miRNAs and the role of exosome-derived miRNAs assumed with diet on the onset and progression of CRC are still limited. In addition, the mixture of different active compounds and their synergism/antagonism and/or additive capacity prompted new studies evaluating the beneficial role of milk-derived compounds in the prevention of CRC, as the most common cancers in the world [40]. To this end, the present study was designed to investigate the profile of exosomal miRNA from buffalo milk and evaluate the biological effects in HCT116 and HT-29 CRC cells, dissecting possible interactions with multiple bioactive components of 3 kDa milk extract (3 kDa milk).

## 2. Materials and Methods

### 2.1. Isolation and Validation miRNA

Total Exosome Isolation (from other body fluids) (4484453, Thermo Fisher Scientific, Waltham, MA, USA) and Total Exosome RNA and Protein Isolation Kit (4478545, Thermo Fisher Scientific, Waltham, MA, USA) were utilized for exosome and small RNA isolation from cow and buffalo milk, following instructions of the manufacturers. The quality of total RNA was checked via NanoDrop2000 spectrophotometer (Thermo Fisher Scientific, Waltham, MA, USA) and then reverse-transcribed into cDNA using Mir-X miRNA first-strand synthesis kit (638315, Takara Bio Inc., Kusatsu, Japan), following the manufacturer′s protocol, on a thermal cycler SureCycler 8800 (Agilent Technologies, Santa Clara, CA, USA). The resulting cDNA was spotted on a pre-designed customized ID3EAL™ PanoramiR miRNA 96-well plate (MiRXES, Singapore, Republic of Singapore), which included qPCR master mixes and Spike-In, and PCR performed by using a CFX96 Real-Time PCR Detection System (Bio-Rad, Hercules, CA, USA). To validate the results of the card, the expression of mature miRNAs was independently determined in both buffalo and cow milk. RNA reverse transcription into cDNA was carried out utilizing ID3EAL cDNA Synthesis System (1103101, MiRXES, Singapore, Republic of Singapore), following the manufacturer′s protocol, on a thermal cycler SureCycler 8800 (Agilent Technologies, Santa Clara, CA, USA) and the following ID3EAL Individual miRNA RT Primer 1-plex: bta-miR-27b (1103111-BTA0000419A, MiRXES, Singapore, Republic of Singapore), bta-miR-148a (1103111-BTA0000243A, MiRXES, Singapore, Republic of Singapore), and bta-miR-15b (1103111-BTA0004586A, MiRXES, Singapore, Republic of Singapore). Quantitative real-time assays were carried out using a CFX96 Real-Time PCR Detection System (Bio-Rad, Hercules, CA, USA) with ID3EAL miRNA qPCR Master Mix (1104202, MiRXES, Singapore, Republic of Singapore) and ID3EAL miRNA qPCR assay as primers: bta-miR-27b (1104101-BTA0000419A, MiRXES, Singapore, Republic of Singapore), bta-miR-148a (1104101-BTA0000243A, MiRXES, Singapore, Republic of Singapore), and bta-miR-15b (1104101-BTA0004586A, MiRXES, Singapore, Republic of Singapore). To normalize total RNA samples, the ID3EAL Complete Spike-in RNA Kit (1102153, MiRXES, Singapore, Republic of Singapore) was used as an appropriate endogenous control. The relative miR expression levels were determined by using the 2^−ΔΔCt^ method (ΔΔ cycle threshold, Ct = (Ct miR–Ct Spike-In) of buffalo milk/(Ct miR–Ct Spike-In) of cow milk), and the data was reported as the mean ± SD of *n* = 3 independent experiments, with each reaction performed in triplicate. 

### 2.2. Milk Extract Preparation and Metabolic Profile

Bulk milk was gathered from Italian Mediterranean dairy buffaloes (*Bubalus bubalis*) originating from industrial buffalo ranches in southern Italy, and 3 kDa extracts were prepared as previously described [10]. Milk samples were centrifuged at 3000× *g* for 15 min at 4 °C to avoid fat globules and, to recover metabolites with low molecular weight, the central aqueous phase was filtered by Amicon Ultra 0.5 mL centrifugal filter with a 3 kDa molecular weight cut-off. The content of L-carnitine, acetyl-L-carnitine, propionyl-L-carnitine, glycine betaine, γ-butyrobetaine, and δ-valerobetaine was assessed in milk extracts as previously described [10,11]. HPLC-ESI-MS/MS analyses were conducted with Agilent LC-MSD SL quadrupole ion trap, in positive multiple reaction monitoring (MRM) detecting the highest MS2 transitions for each compound. The chromatography was carried out isocratically with 0.1% formic acid in water at a flow rate of 100 µL/min and 5 µL volumes of standard or samples were injected. Biomolecules were recognized through their retention times and MS2 fragmentation patterns. By comparing the peak area of each compound highest MS2 fragment with the corresponding standard calibration curve and the mean concentration value of each molecule, expressed in mg/L, the amount of each molecule was ascertained. Each sample was analyzed in triplicate and linearity assessed by correlation coefficients (r2) > 0.99 for each compound. Before being used for cell treatments, milk extracts were sterilized through a 0.22 μm Millipore filter. 

### 2.3. Cell Culture, Transfection, and Treatments

Human colorectal adenocarcinoma HCT116 (CCL-247) and HT-29 (HTB-38) cell lines were obtained from American Type Culture Collection (ATCC, Manassas, VA, USA) and grown as a monolayer in McCoy’s 5A medium (16600-082, Gibco, Life Technologies, Carlsbad, CA, USA) supplemented with 100 U/mL penicillin, 100 mg/mL streptomycin, and 10% fetal bovine serum (FBS, 10270-106, Gibco, Life Technologies, Carlsbad, CA, USA) at 37 °C under a humidified atmosphere with 5% CO_2_. Cells were seeded into multi-well plates the day prior to transfection to promote cell adhesion. HCT116 and HT-29 cells were transfected with 30 nM mimic miR-148a (hsa-miR-148a miRNA Mimic, MCH01336, Applied Biological Materials, Inc. Richmond, BC, Canada), miR-15b (hsa-miR-15b miRNA Mimic, MCH01373, Applied Biological Materials, Inc. Richmond, BC, Canada), miR-27b (hsa-miR-27b miRNA Mimic, MCH01638, Applied Biological Materials, Inc. Richmond, BC, Canada), and miRNA mimic Negative Control (miR-NC, MCH00000, Applied Biological Materials, Inc. Richmond, BC, Canada) was performed, in serum- and antibiotic-free medium, using Lullaby (LL70500, OZ Biosciences, Marseille, France) as transfection reagent. Cells were incubated for 8 h, followed by the addition of FBS. Control (Ctr) cells were treated using the respective maximum volume of Hanks’ balanced salt solution (HBSS)-10 mM Hepes. The transfection efficiency was confirmed by qRT-PCR. Treatments with 3 kDa milk extracts (3 kDa milk) (40% *v*/*v*) for 72 h were performed on CRC cells transfected with mimics (miR-27b^+^) or negative control (miR-NC). To suppress ER-stress, HCT116 and HT-29 cells were incubated for 1 h with 2 μM GSK2606414 (ER-i) to specifically suppress PERK action (protein kinase RNA-like ER kinase) before transfection and milk treatments.

### 2.4. Validation of miRNA Overexpression in Transfected Cells

Total RNA was obtained through the Total RNA purification kit (17200, Norgen Biotek Corp., Thorold, ON, Canada), as indicated in the manufacturer’s protocol, and then quantified by a NanoDrop2000 spectrophotometer (Thermo Fisher Scientific, Waltham, MA, USA). Reverse transcription from RNA to cDNA was obtained by means of the ID3EAL cDNA Synthesis System (1103101, MiRXES, Singapore, Republic of Singapore), following the manufacturer′s protocol, on a thermal cycler SureCycler 8800 (Agilent Technologies, Santa Clara, CA, USA) and the following ID3EAL Individual miRNA RT Primer 1-plex: hsa-miR-27b-3p (1103111-HSA0000419A, MiRXES, Singapore, Republic of Singapore), hsa-miR-148a-3p (1103111-HSA0000243A, MiRXES, Singapore, Republic of Singapore), and hsa-miR-15b-3p (1103111-HSA0004586A, MiRXES, Singapore, Republic of Singapore). Quantitative real-time assays were carried out using a CFX96 Real-Time PCR Detection System (Bio-Rad, Hercules, CA, USA) with ID3EAL miRNA qPCR Master Mix (1104202, MiRXES, Singapore, Republic of Singapore) and ID3EAL miRNA qPCR assay as primers: hsa-miR-27b-3p (1104101-HSA0000419A, MiRXES, Singapore, Republic of Singapore), hsa-miR-148a-3p (1104101-HSA0000243A, MiRXES, Singapore, Republic of Singapore), and hsa-miR-15b-3p (1104101-HSA0004586A, MiRXES, Singapore, Republic of Singapore). The relative miR expression levels were determined by comparing the expression of each miRNA to that of the small-nuclear-U6, chosen as an endogenous control [42], using the 2^−ΔΔCt^ method (ΔΔ cycle threshold, Ct = (Ct miR–Ct U6) of transfected cells/(Ct miR–Ct U6) of control), and the data was reported as the mean ± SD of *n* = 3 independent experiments, with each reaction performed in triplicate.

### 2.5. Viability Assay

Cell Counting Kit-8 (CCK-8 Donjindo Molecular Technologies, Inc., Rockville, MD, USA) was performed to evaluate HCT116 and HT-29 cell viability, following the manufacturer’s indications. After treatments, 10 μL of CCK-8 solution was added to each well and the plate was incubated for 4 h at 37 °C. Cell absorbance was measured at 450 nm with a microplate reader (model 680, Bio-Rad, Hercules, CA, USA) and viability expressed as % of control of *n* = 4 independent experiments.

### 2.6. Apoptosis Detection

Apoptotic cell death was investigated with Annexin V Apoptosis detection kit (556547, BD Pharmigen, Franklin Lakes, NJ, USA), according to manufacturer’s instructions. HCT116 and HT-29 cells were detached with trypsin, washed with phosphate buffered saline (PBS), and incubated for 30 min in 1× binding buffer containing 2 μL Annexin V-FITC and 2 μL propidium iodide (PI) (20 μg/mL). Flow cytometry analysis was performed with a BD Accuri C6 cytometer (BD Biosciences, San José, CA, USA) recording for each sample at least 20,000 events and results evaluated by FlowJo V10 software (Williamson Way, Ashland, OR, USA).

### 2.7. ROS Evaluation

Levels of mitochondrial ROS were measured using MitoSOX Red Mitochondrial Superoxide Indicator (M36008, Invitrogen, Waltham, MA, USA), following manufacturer’s indications. Treated cells were washed with pre-warmed 1× HBSS-Hepes 10 mM and stained for 30 min with 5 µM MitoSOX probe in complete medium. Cells were visualized on a fluorescence microscope EVOS FL Cell Imaging System (Thermo Scientific, Rockford, IL, USA) and fluorescence analyzed through a BD Accuri C6 cytometer (BD Biosciences, San José, CA, USA). At least 20,000 events were analyzed per sample using FLOWJO V10 software (Williamson Way, Ashland, OR, USA). The ROS-inducer menadione (100 µM) (M57405, Sigma Aldrich, St. Louis, MO, USA) was used as positive control, by incubating HCT116 and HT-29 cells at 37 °C for 30 min in the dark before staining with MitoSOX probe. The content of extracellular ROS was established with Amplex Red Hydrogen Peroxide/Peroxidase Assay kit (A22188, Invitrogen, Waltham, MA, USA). According to the manufacturer’s indication, 100 μL Amplex red reagent, containing 50 μM Amplex Red and 0.1 U HRP/mL, was added to 20 μL cell suspension with 2 × 104 live cells in Krebs–Ringer phosphate glucose buffer. After 1 h incubation at 37 °C, the resulting fluorescence was measured at 530 nm as excitation wavelength and 590 nm as emission wavelength with a multiplate reader (model Infinite M200, Tecan, Männedorf, Switzerland). Extracellular ROS levels were quantified using an H_2_O_2_ standard curve. Experiments were performed with *n* = 5 replicates.

### 2.8. Mitochondria Damage Assessment

Damaged mitochondria fused to lysosomes were detected using the Mitophagy Detection Kit (MD01, Dojindo Molecular Technologies, Tokyo, Japan), following the manufacturer’s protocol. HCT116 and HT-29 cells were stained using 100 nM Mtphagy Dye (red) at 37 °C for 30 min and then incubated for additional 30 min with 1 μM Lyso Dye (green). After washing with HBSS-Hepes 10 mM, fluorescent images were captured through EVOS FL Cell Imaging System (Thermo Scientific, Rockford, IL, USA) microscope and fluorescence gained with a BD Accuri C6 cytometer (BD Biosciences, San José, CA, USA). For each specimen, at least 20,000 events were collected, and the analysis was carried by FLOWJO V10 software (Williamson Way, Ashland, OR, USA).

### 2.9. ER-Stress Detection

The occurrence of ER-stress was evaluated by ER-Tracker Blue-White DPX dye (E12353, Invitrogen, Waltham, MA, USA), following manufacturer’s instruction. After treatments, cells were stained for 30 min at 37 °C with 1 µM ER-Tracker probe in HBSS-Hepes 10 mM. Cell images were captured on a fluorescent microscope EVOS FL Cell Imaging System (Thermo Scientific, Rockford, IL, USA), while fluorescence registered using a BD Accuri C6 cytometer (BD Biosciences, San José, CA, USA). For each sample, at least 20,000 events were collected, and the FLOWJO V10 program (Williamson Way, Ashland, OR, USA) was used for analysis. The ER-stress inducer tunicamycin (5 µg/mL) (T7765, Sigma Aldrich, St. Louis, MO, USA) was incubated for 16 h at 37 °C and used as positive control.

### 2.10. Extracellular Calcium Levels

Extracellular calcium content was determined by Calcium assay kit (ab102505, Abcam, Cambridge, UK), following manufacturer’s indications. Briefly, cells were seeded in 96-well plates and, after treatment, chromogenic reagent (90 μL) and calcium assay buffer (60 μL) were added to each well. After 10 min incubation in the dark, the optical density of the chromogen was measured at 575 nm with a microplate reader (model 680, Bio-Rad, Hercules, CA, USA) and extracellular Ca^2+^ levels, reported as mM, extrapolated from the standard curve.

### 2.11. ER-Stress Related mRNA Expression

Total RNA (500 ng) was first retrotranscribed into cDNA and then amplified with MyTaq™ One-Step RT-PCR kit (BIO-65049, Meridian Bioscience Inc., Cincinnati, OH, USA) as directed by the manufacturer. The PCR reactions were carried out using a SureCycler 8800 thermal cycler (Agilent Technologies, Santa Clara, CA, USA) in 25 µL of total reaction volume.

The oligonucleotide primers for human ER-stress markers were as follows: XBP1: F-5′-TTACGAGAGAAAACTCATGGCC-3′ and R-5′-GGGTCCAAGTTTGTCCAGAATGC-3′; PERK: F-5′-GTCCCAAGGCTTTGGAATCTGTC-3′ and R-5′-CCTACCAAGACAGGAGTTCTGG-3′; IRE1: F-5′-CACCTCCACTCCCTCAACAT-3′ and R-5′-CTTCTTGCAGAGGCCAAAGT-3′; ATF6: F-5′-CAGACAGTACCAACGCTTATGCC-3′ and R-5′-GCAGAACTCCAGGTGCTTGAAG-3′; GAPDH: F-5′-AACGGGAAGCTTGTCATCAA-3′ and R-5′-TGGACTCCACGACGTACTCA-3′. Utilizing GAPDH as the reference endogenous gene, all samples were adjusted for relative quantitation by ImageJ software 1.52n version (Wayne Rasband, National Institutes of Health, Bethesda, MD, USA) and expressed as relative gene expression. Each experiment included a negative control without cDNA.

### 2.12. Cell Lysis and Immunoblotting Analysis

Proteins were extracted using lysis buffer (1% NP-40, 0.5% sodium deoxycholate, 0.1% SDS in PBS) with 10 μg/mL aprotinin, leupeptin, and 1 mM phenylmethylsulfonylfluoride. Protein lysates (20–40 μg) were parted through sodium dodecyl sulfate–polyacrylamide gel electrophoresis and transferred to nitrocellulose membranes. Non-specific binding sites were blocked in 1× TBS 1% casein blocker (1610782, Bio-Rad, Hercules, CA, USA) for 1 h at room temperature, and membranes were incubated overnight at 4 °C with specific primary antibodies: anti-BAX (1:500, orb216030, Biorbyt, Cambridge, UK), anti-Bcl-2 (1:500, E-AB-15522, Elabscience Biotechnology Inc., Houston, TX, USA), anti-extracellular-signal regulated kinase (ERK, 1:3000, 66192-1-Ig, Proteintech Group, Inc., Rosemont, IL, USA), anti-phospho-ERK (1:1000, sc-7383, Santa Cruz Biotechnology, Inc., CA, USA), anti-c-Jun *N*-terminal kinase (JNK, 1:1000, sc-7345, Santa Cruz Biotechnology, Inc., CA, USA), anti-phospho-JNK (1:1000, sc-293136, Santa Cruz Biotechnology, Inc., CA, USA), anti-p38 (1:1000, 9218, Cell Signaling Technology, Danvers, MA, USA), anti-phospho-p38 (1: 1000, sc-166182, Santa Cruz Biotechnology, Inc, CA, USA), caspase-12 (1:1000, E-AB-15533, Elabscience Biotechnology Inc., Houston, TX, USA), anti-C/EBP homologous protein (CHOP, 1:1000, E-AB-65670, Elabscience Biotechnology Inc., Houston, TX, USA), anti-X-box binding protein 1 (XBP1, 1:1000, bs-1668R, Bioss Inc., Woburn, MA, USA), anti-protein kinase RNA-like ER kinase (PERK, 1:1000, E-AB-32546, Elabscience Biotechnology Inc., Houston, TX, USA), anti-activating transcription factor 6 (ATF6, 1:1000, orb340705, Biorbyt, Cambridge, UK), anti-α-tubulin (1:5000, E-AB-20036, Elabscience Biotechnology Inc., Houston, TX, USA); anti-actin (1:3000, ab179467, Abcam, Cambridge, UK), and anti-glyceraldehyde-3-phosphate dehydrogenase (GAPDH, 1:2000, ab9485, Abcam, Cambridge, UK). The peroxidase-conjugated secondary antibodies were incubated for 1 h, and subsequently immunocomplexes were evaluated via Excellent Chemiluminescent Substrate kit (E-IR-R301, Elabscience Biotechnology Inc., Houston, TX, USA), on dried membranes, and revealed by ChemiDoc Imaging System and Image Lab 6.0.1 software (Bio-Rad Laboratories, Milan, Italy). The intensity of each band was measured with ImageJ software 1.52n (Wayne Rasband, National Institutes of Health, Bethesda, MD, USA), compared with the loading control signal and represented as arbitrary units (AU).

### 2.13. Statistical Analysis

Data are the mean ± standard deviation (SD) of at least three independent experiments. GraphPad Prism software version 9.1.2 (GraphPad Software Inc, La Jolla, CA, USA) was run to conduct the statistical analyses. The one-way ANOVA and Tukey’s post hoc test were used to find the significance among the variables. A difference was deemed statistically significant if the *p*-value was less than 0.05.

## 3. Results

### 3.1. Buffalo Milk miRNA Content

**Figure 1:**
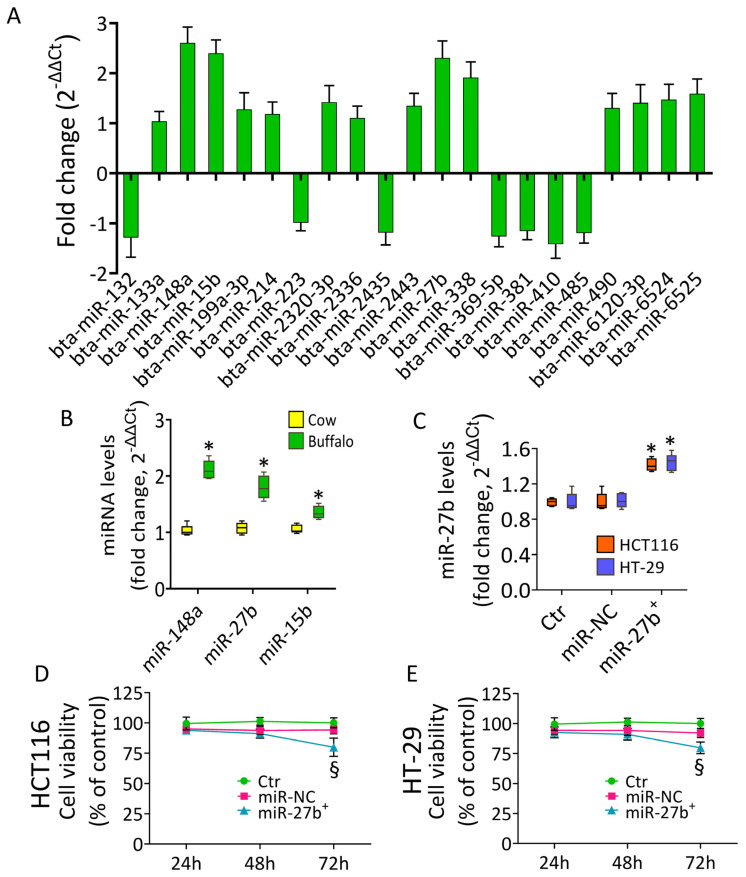
Expression pattern of miRNA in buffalo milk. (**A**) MiRNA expression profile of buffalo milk performed by a 96-well customized card analysis. Twenty-one miRNAs resulted differentially expressed in buffalo milk, compared to control bovine milk, with significant values *p* < 0.05. MiRNA levels are reported as bars representing the mean ± SD. (**B**) The relative expression of milk-derived miR-148a, miR-15b, and miR-27b was analyzed by qRT-PCR and normalized with Spike-In as endogenous control. MiRNA levels are reported as floating bars with line representing the mean ± SD. * *p* < 0.05 vs. cow milk. (**C**) The relative expression of miR-27b, analyzed by qRT-PCR and normalized with U6 as endogenous control, in HCT116 and HT-29 transfected with 30 nM mimic Negative Control (miR-NC) and miR-27b mimic (miR-27b^+^). MiRNA levels are reported as floating bars with a line representing the mean ± SD. Cell Counting Kit-8 assay was performed in order to evaluate the cell viability of (**D**) HCT116 and (**E**) HT-29 cells. The transfection was performed using 30 nM mimic Negative Control (miR-NC) and miR-27b mimic (miR-27b^+^) and viability expressed as % of control of *n* = 4 independent. Control cells (Ctr) were treated with the corresponding highest volume of HBSS-10 mM Hepes. * *p* < 0.05 vs. miR-NC; ^§^ *p* < 0.01 vs. miR-NC.

The expression levels of miRNAs in buffalo milk were investigated with 96-miRNAs customized plate. Results showed that 21 miRNAs were found to have different expression levels compared to control cow milk (*p* < 0.05) (Figure 1A), with miR-148a, miR-27b, and miR-15b being the most upregulated.

Validation of miR-148a, miR-27b, and miR-15b levels, sharing homology score values greater than 100 with human counterparts (https://www.mirbase.org/), accessed on 21 October 2021, confirmed that the relative miRNA expression resulted upregulated in buffalo milk compared to control cow milk, with fold-change values of 2.1-fold for miR-148a, 1.8-fold for miR-27b, and 1.3-fold for miR-15b (*p* < 0.05) (Figure 1B). Given these observations, miR-148a, miR-27b, and miR-15b were further investigated in HCT116 and HT-29 CRC cells.

### 3.2. Cytotoxic Effects of miR-27b

The qRT-PCR analysis proved the transfection efficacy of HCT116 and HT-29 cells with mimic miR-27b (miR-27b^+^), miR-148a (miR-148a^+^), and miR-15b (miR-15b^+^) (*p* < 0.05) (Figure 1C and Supplementary Figure S1). Cell viability assays showed that miR-27b^+^ decreased the CRC cell viability after 72 h of transfection (*p* < 0.01 vs. miR-NC) (Figure 1D,E) and displayed the higher cytotoxic effect compared to miR-148a^+^ and miR-15b^+^ (Supplementary Figure S1). The studies on the cell death mechanism(s) have been performed only on miR-27b^+^ as it displayed a higher cytotoxic effect compared to miR-148a^+^ and miR-15b^+^.

### 3.3. MiR-27b Promotes Mitochondrial Stress and Apoptotic Death

In order to provide whether the cytotoxic effect of miR-27b^+^ was related to the occurrence of mitochondrial oxidative stress, along with lysosome flux and apoptotic cell death, the involvement of these pathways has been assessed.

**Figure 2:**
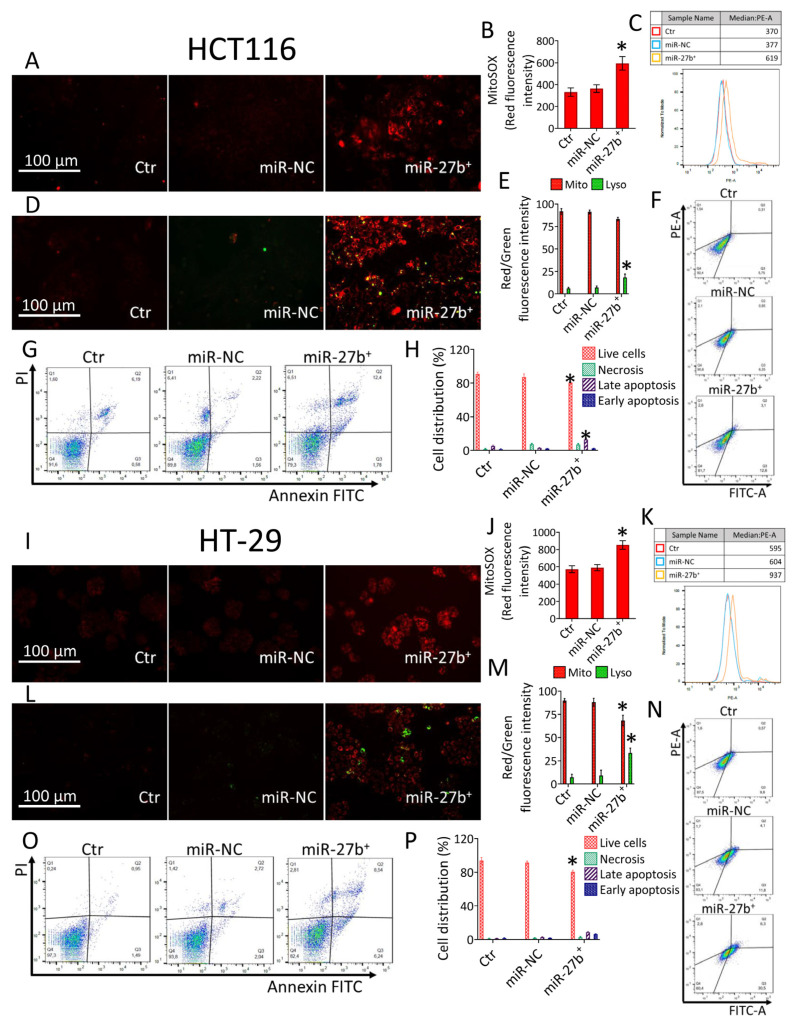
MiR-27b induced mitochondrial stress and apoptosis. (**A**–**C**,**I**–**K**) Representative images by fluorescence microscopy and FACS analysis of mitochondrial ROS detection, (**D**–**F**,**L**–**N**) mitophagy (red) and lysosome (green) dyes, and (**G**,**H**,**O**,**P**) representative dot plots and analyses of annexin V-FITC and propidium iodide (PI)-staining in HCT116 and HT-29 cells transfected for 72 h with 30 nM mimic Negative Control (miR-NC) and miR-27b mimic (miR-27b^+^). Control cells (Ctr) were treated with the highest volume of HBSS-10 mM Hepes. Scale bars = 100 µm. Cell viability/death was assessed by flow cytometry detecting at least 20,000 events. Lower left quadrant: viable cells; upper left quadrant: necrotic cells; lower right quadrant: early apoptotic cells; upper right quadrant: late apoptotic cells. * *p* < 0.05 vs. miR-NC.

MiR-27b^+^ cells showed increased mitochondrial ROS content and lysosome accumulation (*p* < 0.05 vs. miR-NC) in both transfected CRC cell lines (Figure 2).

The ROS promoting menadione was used as positive control (Supplementary Figure S2). Flow cytometry-based annexin V/PI determination showed a decrease of live cells (*p* < 0.05 vs. miR-NC) and apoptosis (*p* < 0.05 vs. miR-NC), while necrotic death was not appreciated (Figure 2).

### 3.4. Milk Increases the Apoptotic Activity in miR-27b^+^ Cells

**Figure 3:**
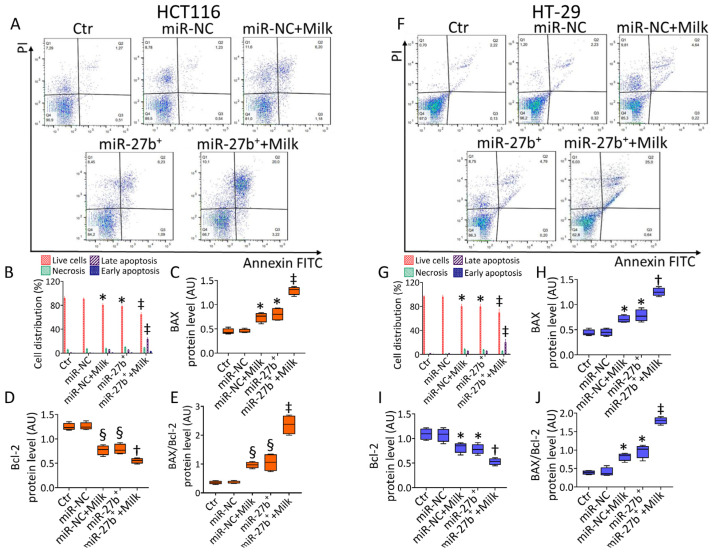
MiR-27b^+^+Milk induced CRC apoptosis. (**A**) Representative dot plots and analyses of annexin V-FITC and propidium iodide (PI)-staining and immunoblotting analyses of Bax/Bcl-2 protein levels in (**A**–**E**) HCT116 and (**F**–**J**) HT-29 cells transfected with 30 nM mimic Negative Control (miR-NC) and miR-27b mimic (miR-27b^+^) or with miR-NC and miR-27b^+^ before 72 h with 40% *v*/*v* milk treatment (miR-NC+Milk and miR-27b^+^+Milk). Control cells (Ctr) were treated with the highest volume of HBSS-10 mM Hepes. Cell mortality and viability were assessed using flow cytometry detecting at least 20,000 events. Lower left quadrant: viable cells; upper left quadrant: necrotic cells; lower right quadrant: early apoptotic cells; upper right quadrant: late apoptotic cells. Western blotting results (*n* = 4) are expressed as arbitrary units (AU). * *p* < 0.05 vs. miR-NC; ^§^ *p* < 0.01 vs. miR-NC; ^†^ *p* < 0.05 vs. miR-27b^+^; ^‡^ *p* < 0.01 vs. miR-27b^+^.

The effect of 3 kDa milk was tested in miR-27b^+^ cells. In line with previous studies, 3 kDa milk showed a content of betaines and short-chain acyl carnitines as follows; 52.3 ± 0.6 mg/L acetyl-L-carnitine, 24.7 ± 0.8 mg/L propionyl-L carnitine, 47.0 ± 0.5 mg/L L-carnitine, 6.3 ± 0.2 mg/L γ-butyrobetaine, 8.1 ± 0.6 mg/L glycine betaine, and 26.4 ± 0.5 mg/L δ-valerobetaine. To explore the antitumor activity of milk in miR-27b^+^ cells, the flow cytometry-based annexin V/PI method and the evaluation of the BAX/Bcl-2 ratio was provided. Results showed that 72 h of treatment with 3 kDa milk 40% (*v*/*v*) resulted in a higher apoptotic effect determining a strong percentage of cells in late apoptosis, along with a lower live cell percentage (*p* < 0.01 vs. miR-27b^+^). On the contrary, no significant necrotic percentage was revealed. (Figure 3).

These results were supported by a higher increase in the BAX/Bcl-2 ratio in miR-27b^+^ + Milk treated cells (*p* < 0.01 vs. miR-27b^+^) (Figure 3C–E,H–J and Supplementary Figure S3).

### 3.5. Milk Exacerbates Mitochondrial ROS Accumulation in miR-27b^+^ Cells

**Figure 4:**
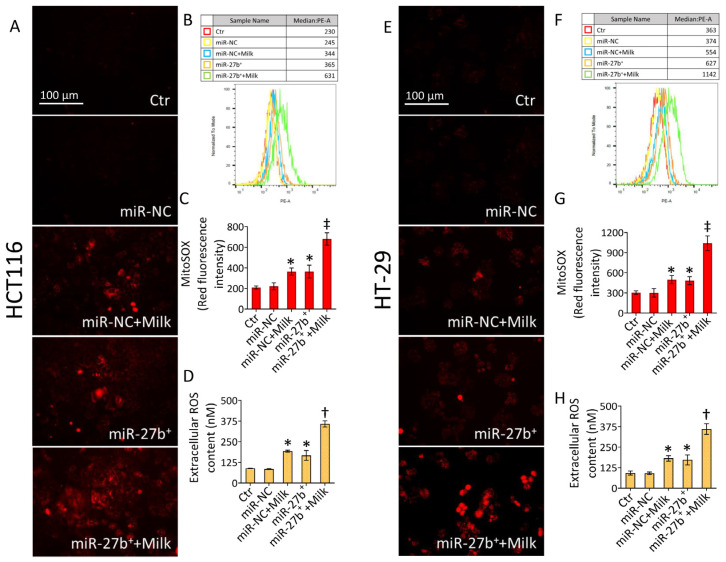
MiR-27b^+^ + Milk provoked oxidative stress. Representative images by fluorescence microscopy and FACS analysis of mitochondrial ROS detection and extracellular ROS content in (**A**–**D**) HCT116 and (**E**–**H**) HT-29 cells transfected with 30 nM mimic Negative Control (miR-NC), miR-27b mimic (miR-27b^+^), with miR-NC and miR-27b^+^ before 72 h with 40% *v*/*v* milk treatment (miR-NC + Milk and miR-27b^+^ + Milk). Control cells (Ctr) were treated with the upper volume of HBSS-10 mM Hepes. Scale bars = 100 µm. * *p* < 0.05 vs. miR-NC; ^†^ *p* < 0.05 vs. miR-27b^+^; ^‡^ *p* < 0.01 vs. miR-27b^+^.

Furthermore, miR-27b^+^ cells showed increased mitochondrial and extracellular ROS levels (*p* < 0.05 vs. miR-NC) (Figure 4).

Treatment of miR-27b^+^ cells with milk determined a more intense oxidative stress, as revealed by mitochondria (*p* < 0.01 vs. miR-27b^+^) and extracellular (*p* < 0.05 vs. miR-27b^+^) ROS accumulation (Figure 4).

### 3.6. Lysosome Accumulation

**Figure 5:**
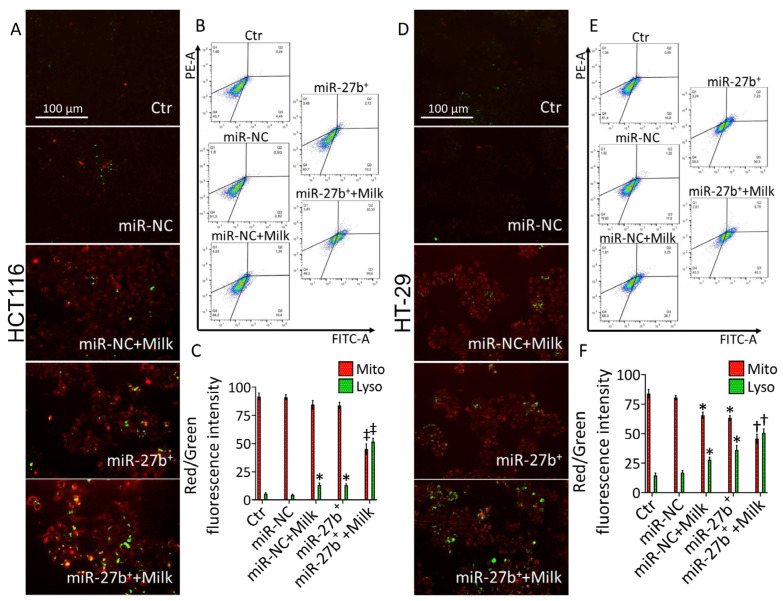
MiR-27b^+^+Milk increased lysosome accumulation. Representative fluorescent images of mitophagy (red) and lysosome (green) dyes and FACS analysis, reported as fluorescence intensity, in (**A**–**C**) HCT116 and (**D**–**F**) HT-29 cells transfected with 30 nM mimic Negative Control (miR-NC) and miR-27b mimic (miR-27b^+^) or with miR-NC and miR-27b^+^ before 72 h with 40% *v*/*v* milk treatment (miR-NC + Milk and miR-27b^+^+Milk). Control cells (Ctr) were treated with the highest volume of HBSS-10 mM Hepes. Scale bars = 100 µm. * *p* < 0.05 vs. miR-NC; ^†^ *p* < 0.05 vs. miR-27b^+^; ^‡^ *p* < 0.01 vs. miR-27b^+^.

Mitochondrial stress is often related to lysosome responses since damaged mitochondria are fused to lysosomes. Evaluation of lysosome accumulation in miR-27b^+^ cells evidenced the occurrence of mitochondrial injury (*p* < 0.05 vs. miR-NC). Moreover, following treatment with milk (miR-27b^+^ + Milk), an increase in green lysosomic-related fluorescent probe was observed, with a more pronounced effect in HCT116 miR-27b^+^ cells (*p* < 0.01 vs. miR-27b^+^) compared to HT-29 miR-27b^+^ cells (*p* < 0.05 vs. miR-27b^+^) (Figure 5).

### 3.7. ER-Stress

**Figure 6:**
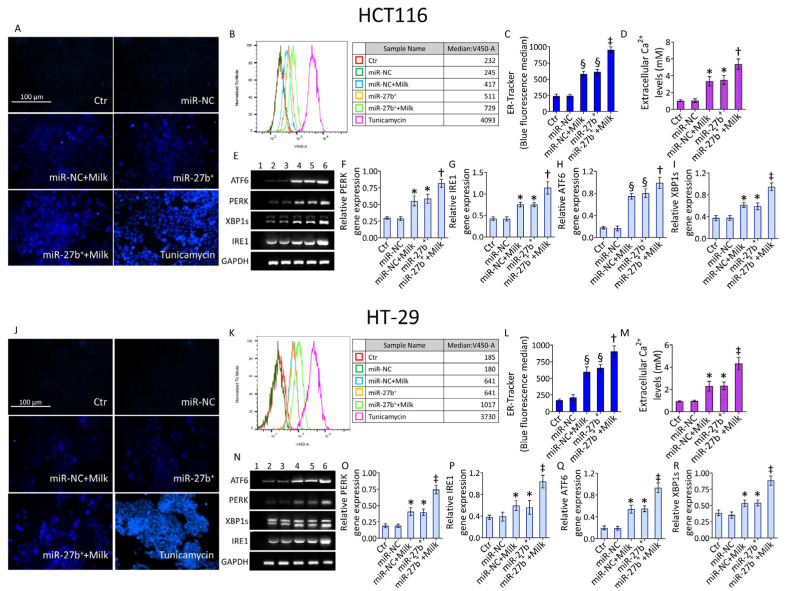
MiR-27b^+^+Milk triggered ER-stress. Representative images by fluorescence microscopy and FACS analysis of ER-tracker dye, expressed as blue fluorescence median, extracellular calcium content, and PERK, IRE1, ATF6, and XBP1s relative mRNA levels assessed in (**A**–**I**) HCT116 and (**J**–**R**) HT-29 cells transfected with 30 nM mimic Negative Control (miR-NC) and miR-27b mimic (miR-27b^+^) or with miR-NC and miR-27b^+^ before 72 h with 40% *v*/*v* milk treatment (miR-NC+Milk and miR-27b^+^+Milk). Control cells (Ctr) were treated with the highest volume of HBSS-10 mM Hepes. Positive control was performed by using the ER-stress inducer tunicamycin (5 µg/mL) for 16 h. Scale bars = 100 µm. Lane 1 = negative control lacking cDNA template; lane 2 = Ctr; lane 3 = miR-NC; lane 4 = miR-NC+Milk; lane 5 = miR-27b^+^; lane 6 = miR-27b^+^+Milk. Levels of mRNA for PCR were adjusted to the GAPDH endogenous control and calculated using ImageJ software. The predicted sizes of the amplified transcripts are visualized on 2.0% agarose gels. * *p* < 0.05 vs. miR-NC; ^§^ *p* < 0.01 vs. miR-NC; ^†^ *p* < 0.05 vs. miR-27b^+^; ^‡^ *p* < 0.01 vs. miR-27b^+^.

Aberrant lysosome accumulation results in excessive ER stress and the related unfolded protein response (UPR). When investigating the effect of miR-27b^+^ on ER-stress and the related UPR, results showed the ER-stress induction (*p* < 0.01 vs miR-NC), as well as upregulated levels of extracellular calcium in HCT116 cells (*p* < 0.05 vs miR-NC) (Figure 6A–D).

For both phenomena, the treatment of miR-27b^+^ cells with milk determined a more consistent effect (*p* < 0.01 vs. miR-27b^+^) (Figure 6A–D). Similarly, more pronounced effects on ER-tracker fluorescence and extracellular calcium levels were found in HT-29 cells miR-27b^+^ treated with milk (*p* < 0.01 vs. miR-27b^+^) (Figure 6J–M). These results were confirmed by mRNA levels of pivotal ER-stress markers, showing that treatment of miR-27b^+^ with milk upregulated mRNA levels of PERK (*p* < 0.01 vs. miR-27b^+^), inositol requiring enzyme 1 (IRE1) (*p* < 0.01 vs. miR-27b^+^), activating transcription factor 6 (ATF6) (*p* < 0.01 vs. miR-27b^+^), and its target X-box binding protein 1 (XBP1) (*p* < 0.01 vs. miR-27b^+^) (Figure 6 and Supplementary Figure S4). The ER inducer tunicamycin was tested as positive control.

### 3.8. Modulation of ER-Stress Markers

**Figure 7:**
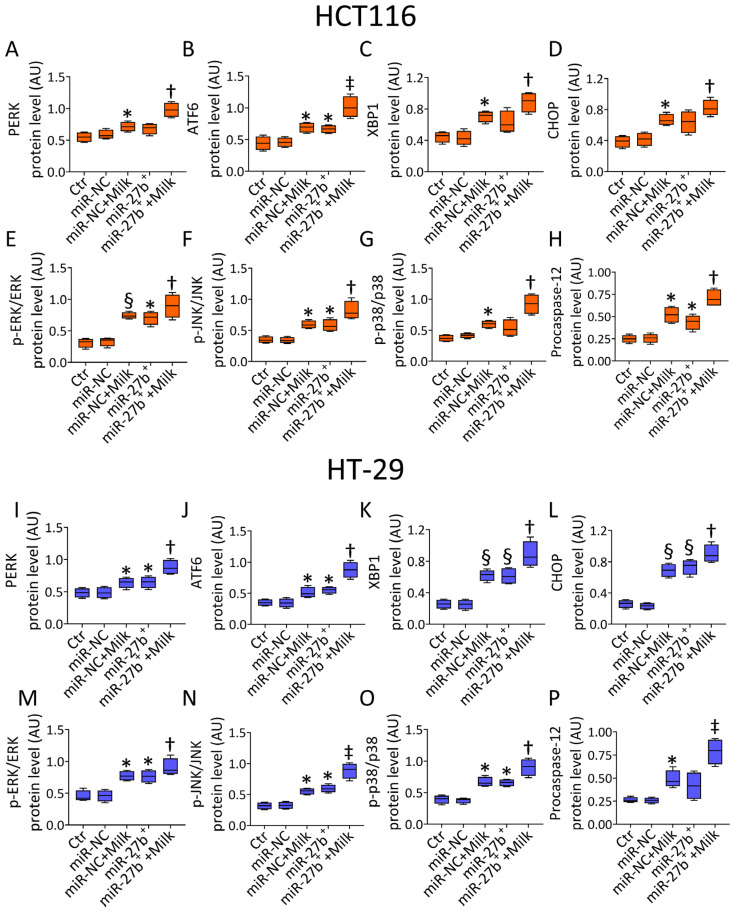
MiR-27b^+^+Milk modulated ER-stress markers. Representative immunoblotting analysis of PERK, ATF6, XBP1, CHOP, phospho-ERK/ERK, phospho-JNK/JNK, phospho-p38/p38, and procaspase-12 in (**A**–**H**) HCT116 and (**I**–**P**) HT-29 cells transfected with 30 nM mimic Negative Control (miR-NC) and miR-27b mimic (miR-27b^+^) or with miR-NC and miR-27b^+^ before 72 h with 40% *v*/*v* milk treatment (miR-NC + Milk and miR-27b^+^ + Milk). Control cells (Ctr) were treated with the corresponding highest volume of HBSS-10 mM Hepes. Results (*n* = 4) are expressed as arbitrary units (AU). * *p* < 0.05 vs. miR-NC; ^§^ *p* < 0.01 vs. miR-NC; ^†^ *p* < 0.05 vs. miR-27b^+^; ^‡^ *p* < 0.01 vs. miR-27b^+^.

To support the unbalance of ER triggered in miR-27b^+^ cells, the expression levels of UPR key proteins and their downstream enzymes were evaluated (Figure 7).

Results showed the upregulation of the expression levels of PERK, ATF6, XBP1, and CCAAT-enhancer-binding homologous protein (CHOP) (*p* < 0.01 vs. miR-NC), with a higher effect in miR-27b^+^+Milk (*p* < 0.05 vs. miR-27b^+^) (Figure 7 and Supplementary Figure S5). MiR-27b^+^ cells also showed a positive modulation of the phosphorylated forms of ERK, JNK, and p38 MAPKs (*p* < 0.05 vs. miR-NC), potentiated by milk treatment (*p* < 0.05 vs. miR-27b^+^) (Figure 7 and Supplementary Figure S5). Moreover, an increase in procaspase-12 expression in miR-27b^+^+Milk cells occurred (*p* < 0.01 vs. miR-27b^+^) (Figure 7 and Supplementary Figure S5).

### 3.9. ER-i Decreases Apoptosis Induced in miR-27b^+^

**Figure 8:**
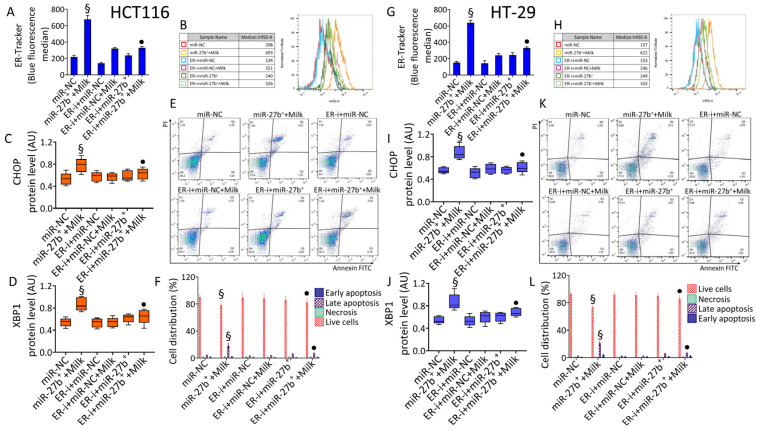
ER-i blocked the apoptotic effect of miR-27b^+^+Milk. Representative FACS analysis of ER-tracker dye, expressed as blue fluorescence median, immunoblotting analysis of CHOP and XBP1, and dot plots and analyses of annexin V-FITC and propidium iodide (PI)-staining in (**A**–**F**) HCT116 and (**G**–**L**) HT-29 cells transfected with 30 nM mimic Negative Control (miR-NC) and miR-27b mimic before 72 h with 40% *v*/*v* milk treatment (miR-27b^+^ + Milk) or incubated for 1 h with 2 μM GSK2606414 (ER-i) and then transfected with miR-NC (ER-i + miR-NC) or with miR-27b^+^ (ER-i+miR-27b^+^) before milk incubation (ER-i+miR-NC+Milk and ER-i+miR-27b^+^+Milk). Cell viability/death was assessed by flow cytometry acquiring at least 20,000 events. Lower left quadrant: viable cells; upper left quadrant: necrotic cells; lower right quadrant: early apoptotic cells; upper right quadrant: late apoptotic cells. Western blotting results (*n* = 4) are expressed as arbitrary units (AU). ^§^ *p* < 0.01 vs. miR-NC; • *p* < 0.01 vs. miR-27b^+^+Milk.

To support the miR-27b^+^ + Milk capacity in triggering apoptosis with concomitant ER stress induction, incubation with ER-i was performed. Treatment with ER-i decreased the ER-stress in miR-27b^+^ cells and opposed the upregulation of CHOP and XBP1 expression in the presence of milk treatment (*p* < 0.01 vs. miR-27b^+^+Milk) (Figure 8 and Supplementary Figure S6).

As well, miR-27b^+^ treated with ER-i and milk (ER-i+miR-27b^+^+Milk) showed lower apoptosis, with an increased viability rate and reduced apoptotic population (*p* < 0.01 vs. miR-27b^+^+Milk) (Figure 8), supporting the possible role of miR-27b in promoting cell death by impairing mitochondrial function and triggering ER-stress.

## 4. Discussion

The results of this study provided new knowledge on exosome-derived miRNA profile from buffalo milk, showing the differential expression of twenty-one miRNAs with miR-148a, miR-15b, and miR-27b as the most up-regulated. At cellular level, mimic experiments with miR-27b demonstrated its anticancer activities in HCT116 and HT-29 cells.

Milk contains different exosome-specific proteins, lipids, bioactive compounds, mRNAs, and non-coding miRNAs, crucial mediators of pivotal biological functions [43,44,45,46]. Bioactive compounds of milk, especially buffalo milk, have been described as epi-nutrient for cancer prevention. In SW480 and SW620 CRC cells δ-valerobetaine, a constitutive milk metabolite with antioxidant, anti-inflammatory, and anti-diabetic activities, impaired cell viability in a time-dependent way, blocked cell cycle at G2/M increasing the expression of cyclin A and cyclin B protein [14,17,47]. Caspase-3 activation, together with loss in mitochondrial membrane potential and SIRT3 downmodulation, enhanced apoptosis. Additional studies carried out on PINK1/Parkin pathways, as upregulation of PINK1, Parkin, and LC3B protein levels, showed the induction of mitochondrial apoptosis [17]. In LoVo cells bioactive betaine promoted cell cycle arrest, upregulation of p21, cyclin A, cyclin B1, and p53 protein expressions and increased necrosis [16]. In the present study, mimic miR-27b induced lysosome accumulation, calcium ions release, and ER-stress in HT-29 and HCT116 cells, through the increase of mRNA and protein levels of the spliced form of XBP1, PERK, IRE1, ATF6, and CHOP, one of the major pathways induced by ROS generation that moves to ER stress and mediates apoptosis [48]. In line with previous observations, results indicated that the effects of mimic miR-27b were linked to the UPR via ER-stress induction and apoptosis of HT-29 and HCT116 cells. The ER-stress response is part of adaptive mechanisms involved in cell survival. However, under severe and prolonged stress conditions, ER unbalance activates apoptotic pathways [49,50]. Several tumors are characterized by a persistent ER stress activation, capable to regulate multiple pro-tumoral cellular mechanisms [51]. Previous evidence underlined Bax/Bak-mediated mitochondrial apoptosis involvement in PERK-mediated ER stress [52]. Moreover, aberrant induction of ER stress markers and their downstream signaling pathways, as MAPK, have emerged as key regulators of tumor growth, aggressiveness, and therapy responsiveness [51]. C-Jun *N*-terminal kinase (JNK), extracellular signal-regulated kinase 1 and 2 (ERK1/2), and p38 are the three principal MAPKs regulating multiple cellular processes, including survival, proliferation, ROS generation, and cell death [53]. The MAPK signaling pathways, including the activation ERK, JNK, and p38 protein levels, are also correlated to UPR activation [54,55,56]. Of interest, our data showed that the treatment with 3 kDa milk enhanced the apoptosis in miR-27b^+^ cells and promoted procaspase-12 upregulation, a pivotal enzyme in the ER-specific apoptosis [57,58,59]. Furthermore, in miR-27b^+^ cells treated with 3 kDa milk, ER-stress and apoptosis were aggravated by the accumulation of phosphorylated JNK, ERK, and p38 protein levels, suggesting a common pathway activated by miR-27b and 3 kDa milk components. Given the evolving evidence that miRNAs are involved in the onset and progression of several diseases, including CRC, miRNA-based therapies are increasing [60]. Over the years, enormous interest and uninterrupted effort of the scientific community has resulted in miRNA-based therapeutics using miRNA mimics to restore specific gene expression and function and/or anti-miRNAs (antagomirs) to counteract the activity of upregulated miRNAs responsible for disease [61]. Therefore, miRNA mimics and antagomirs are highly sought-after therapeutic strategies for modulation of miRNA levels [62]. However, carrier vehicles that take safe and proficient the delivery of miRNA to target tissues are critical for the application of miRNA-based therapies [62]. Indeed, the intrinsic stability of nucleotide sequence and composition of dietary miRNAs affect their absorption [62]. In particular, the susceptibility to degradation by nucleases, rapid clearance from blood, immunotoxicity, and low tissue permeability of food-derived miRNA has been improved by virus-based and non-viral-based miRNA and anti-miRNA delivery systems to maximize the miRNA therapeutic efficacy [62]. Several oligonucleotide carriers have been developed to enhance stability and improve tissue penetration. In vivo viral and non-viral delivery miRNA methods and strategies to circumvent them for a multitude of diseases, with a focus on cancer therapy, have been extensively reviewed [63]. Previous studies described the relation between miR27b expression and CRC development, revealing the miR ability to regulate different pathways through multiple targets. MiR-27b overexpression inhibited tumor growth, cell adhesion, and invasion by modulating ARFGEF1 and paxillin/c-Src axis, in HCT116 cells, and suppressed proliferation and migration in cancer stem cells by downregulating phospho-PI3K p110α and phospho-Akt expression [64,65]. In vitro and in vivo studies showed the miR-27b capacity to sensitize colorectal cancer cells to oxaliplatin by acting on c-Myc/ATG10 chemoresistance signaling pathway [66,67]. In LoVo and HT-29 cells, 3 kDa milk induced variation in mitochondrial stability initiated by excessive ROS accumulation [16]. Of note, when we investigated the role of 3 kDa milk in the miR-27b^+^ cell death mechanism(s), results showed that it enhances the apoptosis and ER-stress induction, in line with previous studies showing synergism among milk bioactive compounds [15,68,69,70]. In addition, as for lactose intolerant consumers, while previous studies reported that ultrafiltration membrane technology decreased the lactose content of milk [71], a complete depletion of lactose can be achieved by enzymatic approach. Further studies using miRNA depleted milk could certainly strengthen the biological role of these small RNAs taken with the diet, define the specific molecular mechanism by which they act, as well as unveil the affected gene expression in CRC cells. Indeed, previous studies conducted in mice models reported that a 4-week diet with miRNA-depleted milk determined a decreased plasma concentration of miR-29b [72]. Although miRNAs are encapsulated in milk exosomes which protect against degradation and facilitate uptake by endocytosis, to date, the potential health benefits of buffalo milk exosome-miRNA are still little explored and offer new perspectives for the interpretation of the roles of dietary miRNA with respect to health and CRC.

## 5. Conclusions

Overall, our work unveils the bioactive role of milk exosomal miR-27b in CRC apoptosis and ER stress and supports the “epi-functional” properties of buffalo milk as a result of the action of multiple components, rather than a single component. Overall, this study provided new insights into the chemo-preventive properties of milk that give this food an ever-increasing role as a health-promoting food to be considered in preventive or adjuvant nutrition programs in CRC.

## Data Availability

The data presented in this study are available from the corresponding author upon request.

## Supplementary Materials

The following supporting information can be downloaded at: https://www.mdpi.com/article/10.3390/nu14235081/s1, Figure S1: Milk-derived miRNA cytotoxicity, Figure S2: Oxidative stress controls, Figure S3: Apoptotic markers, Figure S4: mRNA levels of ER-stress markers, Figure S5: ER-stress markers, Figure S6: ER-i effects.
