# Supplementary material for: Milk Exosomal miR-27b Worsen Endoplasmic Reticulum Stress Mediated Colorectal Cancer Cell Death
Source: Nutrients. 2022 Nov 29;14(23):5081. doi: 10.3390/nu14235081 (PMC9737596; doi:10.3390/nu14235081)
Supplement: Supplementary file 1 [file nutrients-14-05081-s001.zip › nutrients-2053049-supplementary.pdf]

nutrients MDPI

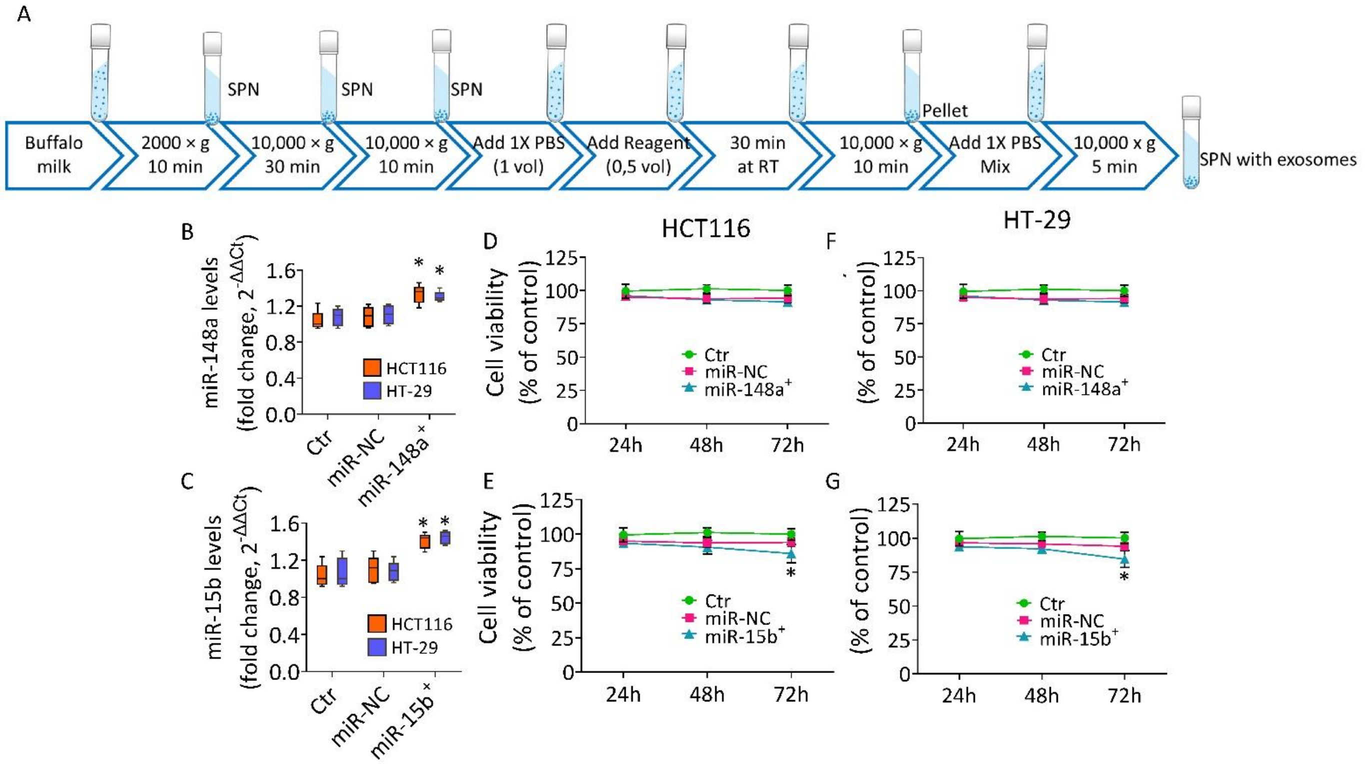

Supplamentary Figure S1: Milk-derived miRNA cytotoxicity. (**A**) Flow chart showing milk exosome miRNA isolation and characterization. The relative expression of (**B**) miR-148a and (**C**) miR-15b, analyzed by qRT-PCR and normalized with U6 as endogenous control, in HCT116 and HT-29 transfected with 30 nM mimic Negative Control (miR-NC), miR-148a mimic (miR-148a+), and miR-15b mimic (miR15b+). MiRNA levels are reported as floating bars with a line representing the mean ± SD. (**D**,**E**) HCT116 and (**F**,**G**) HT-29 cells were transfected with 30 nM mimic Negative Control (miR-NC), miR-148a mimic (miR-148a+), and miR-15b mimic (miR15b+) and cell viability, assessed using Cell Counting Kit-8 assay, expressed as % of control of n = 4 independent. Control cells (Ctr) were treated with the corresponding highest volume of HBSS-10 mM Hepes. *$p < 0.05$ vs. miR-NC.

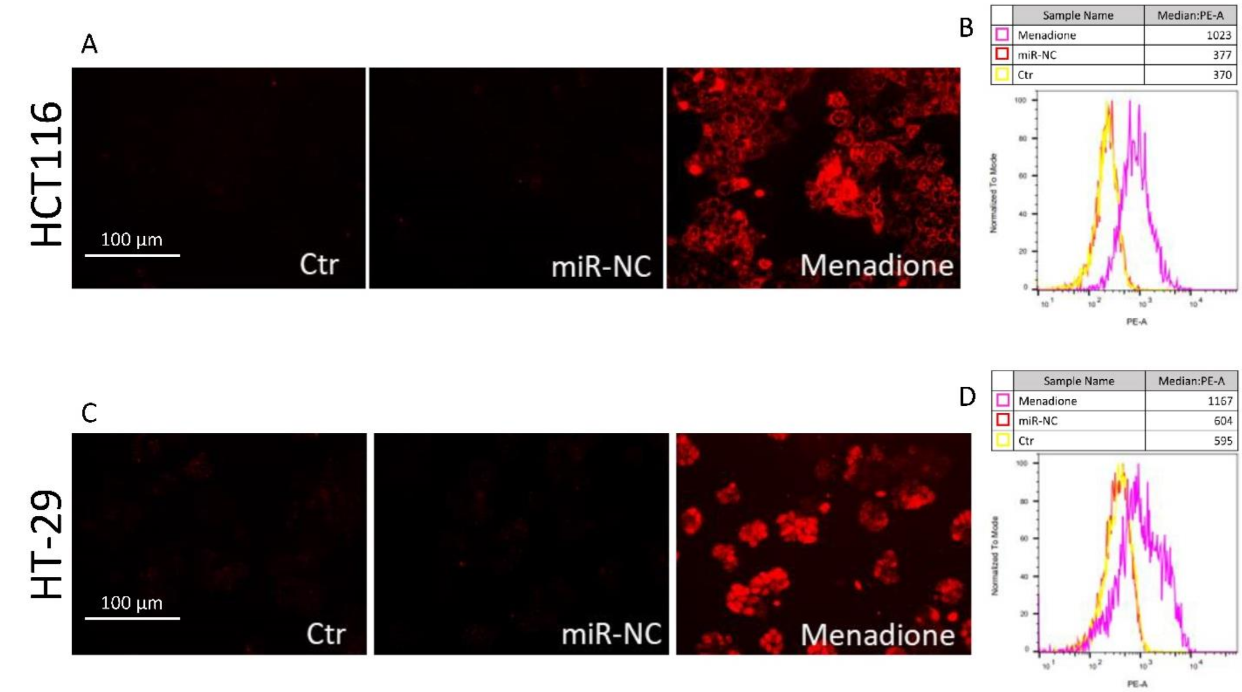

Supplementary Figure S2: Oxidative stress controls. Representative images and cytometer analyses, expressed as red fluorescence median, of mitochondrial ROS detection in (**A**,**B**) HCT116 and (**C**,**D**) HT-29 transfected with 30 nM mimic Negative Control (miR-NC) or treated for 1h with the ROS inducer menadione (100 µM). Control cells (Ctr) were treated with the corresponding highest volume of HBSS-10 mM Hepes. Scale bars = 100 µm.

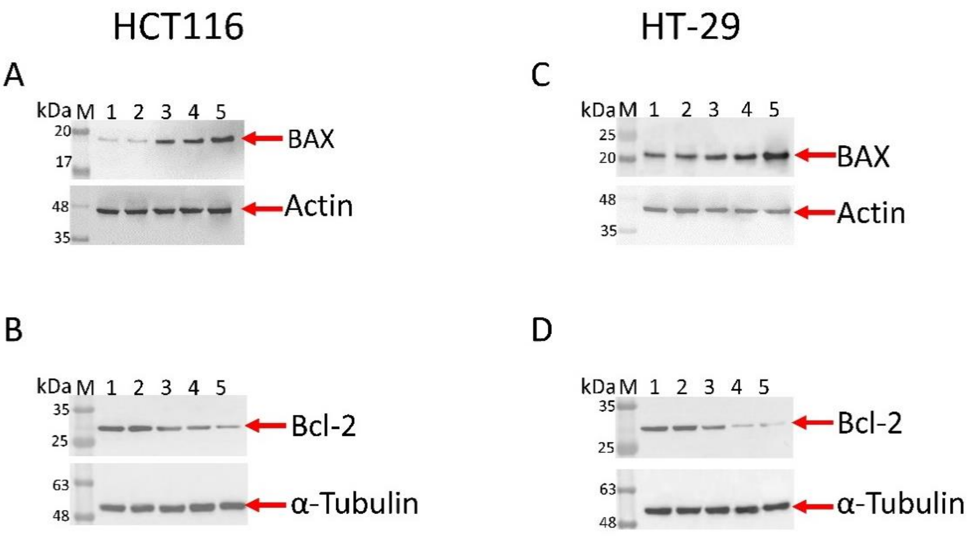

Supplementary Figure S3: Apoptotic markers. Representative cropped blots of BAX, Bcl-2 and their internal controls (actin and α-tubulin) in (**A**,**B**) HCT116 and (**C**,**D**) HT-29 cells transfected with 30 nM mimic Negative Control (miR-NC) and miR-27b mimic (miR-27b^{+}) or with miR-NC and miR-27b^{+} before 72h with 40% v/v milk treatment (miR-NC+Milk and miR-27b^{+}+Milk). Control cells (Ctr) were treated with the corresponding highest volume of HBSS-10 mM Hepes. M = weight markers; Lane 1 = Ctr; lane 2 = miR-NC; lane 3 = miR-NC+Milk; lane 4 = miR-27b^{+}; lane 5 = miR-27b^{+}+Milk.

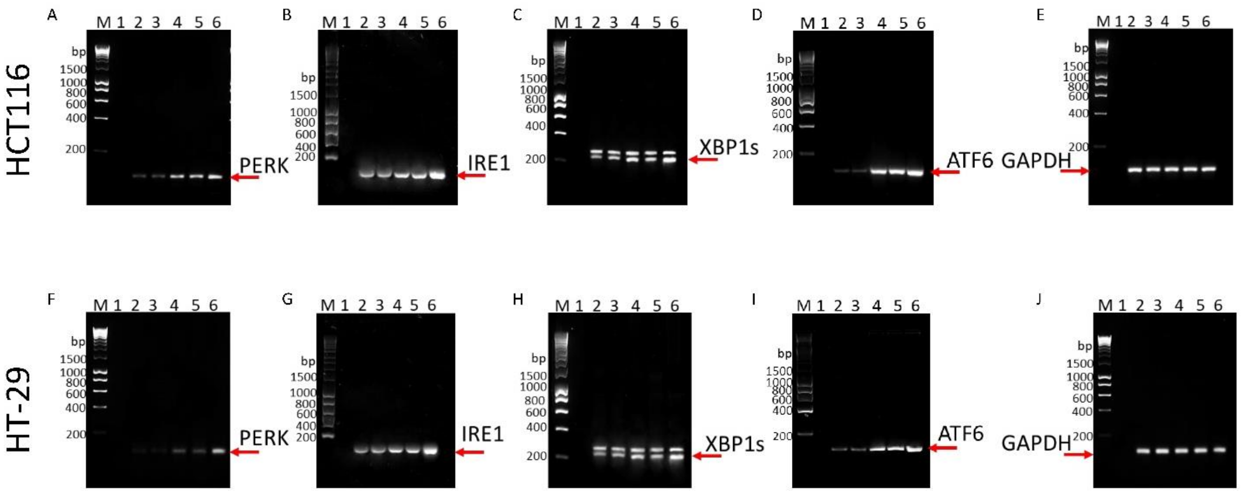

Supplementary Figure S4: mRNA levels of ER-stress markers. Representative PCR products of GAPDH (internal control), PERK, XBP1s, IRE1, and ATF6 in (**A–E**) HCT116 and (**F–J**) HT-29 cells transfected with 30 nM mimic Negative Control (miR-NC) and miR-27b mimic (miR-27b⁺) or with miR-NC and miR-27b⁺ before 72h with 40% v/v milk treatment (miR-NC+Milk and miR-27b⁺+Milk). Control cells (Ctr) were treated with the corresponding highest volume of HBSS-10 mM Hepes. M = molecular markers; Lane 1 = negative control lacking cDNA template; Lane 2 = Ctr; lane 3 = miR-NC; lane 4 = miR-NC+Milk; lane 5 = miR-27b⁺; lane 6 = miR-27b⁺+Milk. The amplified transcripts are shown with the expected sizes on 2.0% agarose gels.

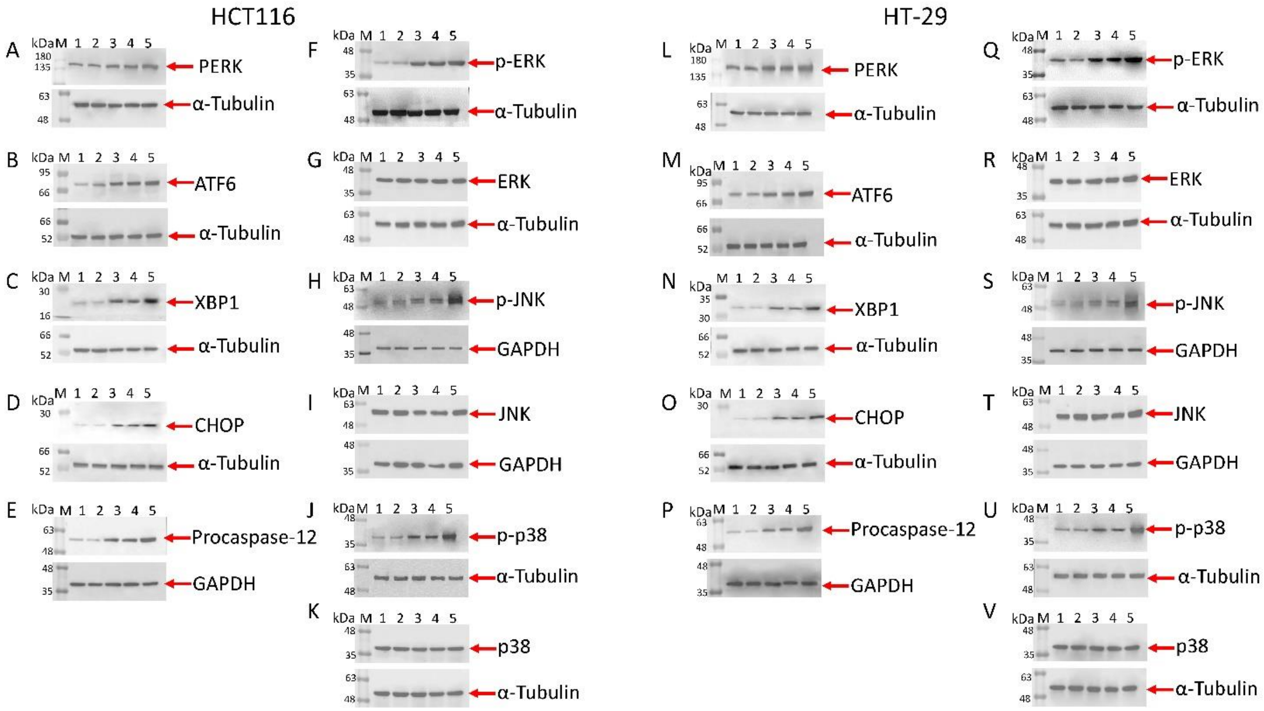

Supplementary Figure S5: ER-stress markers. Representative cropped blots of PERK, ATF6, XBP1, CHOP, procaspase-12, phospho-ERK, ERK, phospho-JNK, JNK, phospho-p38, p38, and their internal controls (GAPDH and α-tubulin) in (**A–K**) HCT116 and (**L–V**) HT-29 cells transfected with 30 nM mimic Negative Control (miR-NC) and miR-27b mimic (miR-27b⁺) or with miR-NC and miR-27b⁺ before 72h with 40% v/v milk treatment (miR-NC+Milk and miR-27b⁺+Milk). Control cells (Ctr) were treated with the corresponding highest volume of HBSS-10 mM Hepes. M = weight markers; Lane 1 = Ctr; lane 2 = miR-NC; lane 3 = miR-NC+Milk; lane 4 = miR-27b⁺; lane 5 = miR-27b⁺+Milk.

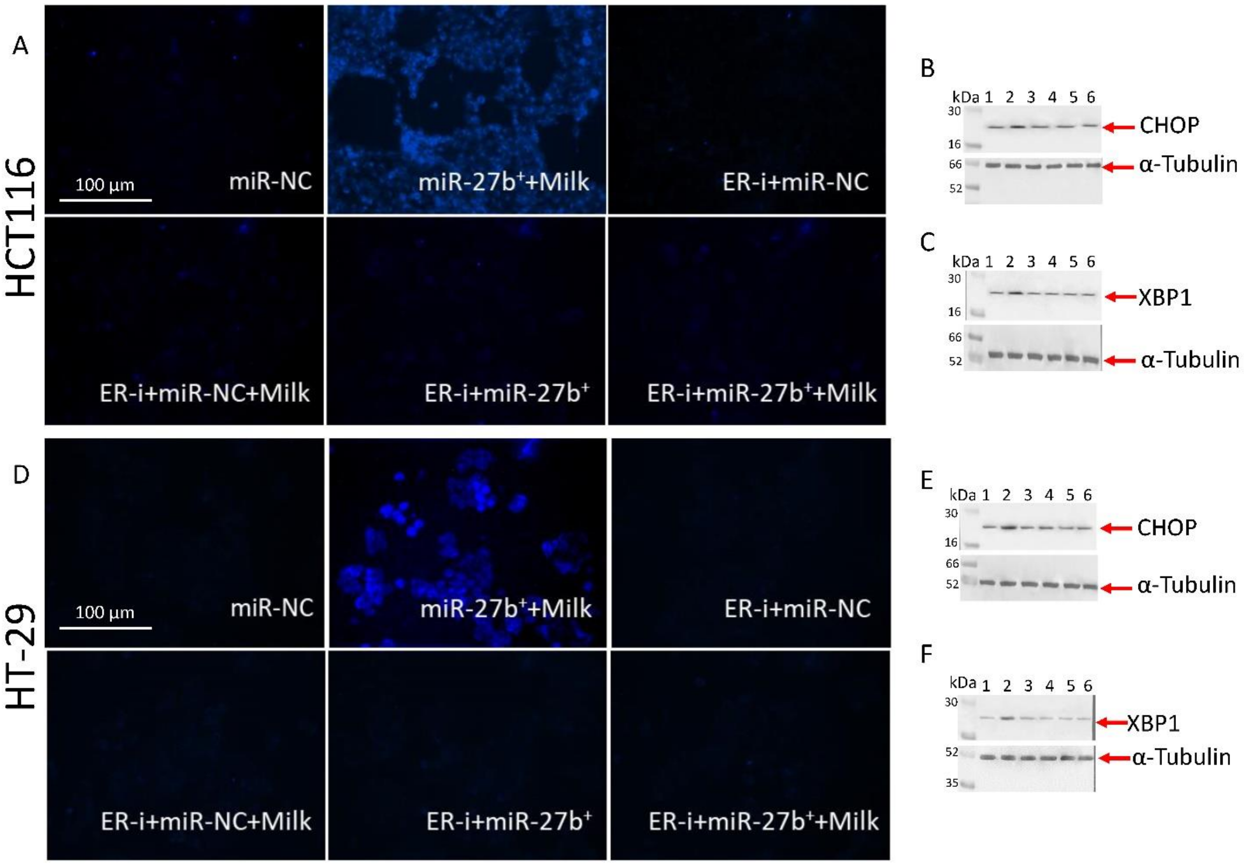

Supplementary Figure S6: ER-i effects. Representative images by fluorescence microscopy and cropped blots of CHOP, XBP1, and the internal control (α-tubulin) in (**A–C**) HCT116 and (**D–F**) HT-29 cells transfected with 30 nM mimic Negative Control (miR-NC) and miR-27b mimic before 72h with 40% v/v milk treatment (miR-27b$^+$+Milk) or incubated for 1h with 2 µM GSK2606414 (ER-i) and then transfected with miR-NC (ER-i+miR-NC) or with miR-27b$^+$ (ER-i+ miR-27b$^+$) before milk incubation (ER-i+miR-NC+Milk and ER-i+miR-27b$^+$+Milk). Scale bars = 100 µm. M = weight markers; Lane 1 = miR-NC; lane 2 = miR-27b$^+$+Milk; lane 3 = ER-i+miR-NC; lane 4 = ER-i+ miR-NC+Milk; lane 5 = ER-i+miR-27b$^+$, lane 6 = ER-i+miR-27b$^+$+Milk.
